# The effects of meditation on individuals facing loneliness: a scoping review

**DOI:** 10.1186/s40359-021-00585-8

**Published:** 2021-05-22

**Authors:** Gurleen K. Saini, Saud B. Haseeb, Zhala Taghi-Zada, Jeremy Y. Ng

**Affiliations:** Department of Health Research Methods, Evidence, and Impact, Faculty of Health Sciences, McMaster University, Michael G. DeGroote Centre for Learning and Discovery, Room 2112, 1280 Main Street West, Hamilton, ON L8S 4K1 Canada

**Keywords:** Loneliness, Meditation, Mind–body medicine, Mindfulness, Scoping review

## Abstract

**Background:**

Meditation is defined as a mind and body practice focused on interactions between the brain, mind, body, and behaviour, containing four key elements: a quiet location with little distractions, a comfortable posture, a focus of attention, and an open attitude. We sought to review the benefits of meditation on the alleviation of loneliness.

**Methods:**

A scoping review was conducted based on Arksey and O’Malley’s five-stage framework. Eligibility criteria included primary studies of any type that investigated the effects of meditation on loneliness. Search strategies were developed and conducted on MEDLINE, EMBASE, AMED, and CINAHL. The National Center for Complementary and Integrative Health, and American Psychological Association websites were also searched. Articles meeting the inclusion criteria were critically reviewed using a descriptive-analytical narrative method.

**Results:**

Thirteen studies met our inclusion criteria and were published between 2012 and 2020 across 10 countries. Eleven studies reported improvements in relation to loneliness. Of the remaining two studies (15%), one mentioned the alleviation of loneliness, but only looked primarily at social closeness in lonely individuals. The other study found a correlation between loneliness and nuclear factor (NF)-κB levels, which was the measured outcome; however, the direct effects of meditation on loneliness were unclear. Three main themes emerged from our analysis, as follows: 1) positive results across all studies, 2) relatively small randomized control trials conducted over the last decade, and 3) lack of diverse demographic information.

**Conclusions:**

While a small number of studies exist at this intersection, given all included studies indicated positive findings, the effects of meditation in alleviating loneliness are promising. Future research should be directed at understanding how meditation mitigates loneliness and how this intervention can impact practice for healthcare professionals.

## Background

Meditation is defined as a mind and body practice focused on interactions between the brain, mind, body, and behaviour, containing four key elements: a quiet location with little distractions, a comfortable posture, a focus of attention, and an open attitude [1]. Meditation is often used for its various health benefits, specifically the alleviation of certain mental states, such as loneliness [2]. While meditation has become popular in Western countries and has been a staple in many Eastern cultures, including Buddhism, due to its positive benefits on mental health, these benefits are not clearly defined in many cases [3, 4].

Over recent years, an increasing number of clinically relevant studies have been conducted in the field of meditation, shedding light on its effects on various mechanisms, specifically the neurobiological mechanisms called S-ART [5]. This refers to self-awareness, self-regulation, and self-transcendence [5]. Meditative practices overall focus on navigating difficult emotions and feelings [6]. Mindfulness practices, such as loving-kindness meditation specifically, which helps to create unconditional kind attitudes towards others and oneself [7], has been shown by Hutcherson et al. [8] to increase feelings of social connectedness and consequently decrease feelings associated with social isolation. Therefore, mindfulness meditation training can be a successful tool to implement in order to reduce feelings of isolation due to the downregulation of the expression of inflammation-related genes, which are parallel to reductions in loneliness [9].

While there is no universal definition for the term ‘loneliness’, it is commonly defined as a state of solitude or being alone [10]. Loneliness can also be defined as the perception of being alone, which constitutes having a negative state of mind associated with deficient social relations rather than actually being alone, due to the various forms of loneliness that exist: (1) chronic loneliness and (2) reactive loneliness [10, 11].

Currently, loneliness is likely to be exacerbated as a result of the coronavirus disease 2019 (COVID-19) pandemic, due to a decrease in regular modes of communication, such as intimate interactions and face-to-face contact, as well as due to a more vulnerable aging population, who, under normal circumstances, disproportionately face loneliness [12, 13]. Before the World Health Organization declared the COVID-19 pandemic [14], the prevalence rates of loneliness and social isolation were already known to be between 10–40% across the United States, China and various European countries, that it was being described as a “behavioral epidemic” [15]. This was specifically being seen in older adults, with both conditions co-occurring frequently [15]. The susceptibility of loneliness in older adults is due to various factors, such as living alone or a lack of familial connections, reduced connections to one’s culture of origin, loss of friendship networks and associated problems in creating new ones, among others [13].

During the beginning of the pandemic between January and May of 2020, there had already been a statistically significant increase in loneliness (interaction-*p* = 0.018) measured based upon a three-item Loneliness Scale, which included the 3 following items: feeling “that you lack companionship”, “left out”, and “isolated from others” [16]. This was most often associated with a reduction in social supports, resulting from the pandemic-associated restrictions that were implemented [16]. As such, negative behavioural health impacts associated with the pandemic posed a threat of worsening after the initial outbreak, including loneliness, which can cause increases in depressive symptoms [16]. While it is challenging to fully identify the extent to which this has occurred to date, evidence exists suggesting that lockdown policies and self-isolation protocols have been found to have negative impacts on the levels of loneliness experienced by individuals both during and after lockdown [17]. Due to the increasing public health issue of loneliness and its effects on health and well-being, alleviation of feelings of loneliness through therapies of value can result in positive health outcomes, such as a lower risk for physiological dysregulation and inflammation [18]. The pandemic has also brought with it an increase in the usage of meditation apps [19]. While it is known that loneliness causes adverse health outcomes and meditation has been linked to its alleviation, the sum benefits identified by research remain unknown. To date, the quantity and type of studies investigating the effects of meditation on loneliness has not been indicated by the literature. Therefore, this was the purpose of this study, using a scoping review methodology.

## Methods

### Approach

Scoping reviews are used to review a body of literature and are defined as studies that aim to map the literature on a particular topic or research area and provide an opportunity to identify key concepts; gaps in the research; and types and sources of evidence to inform practice, policymaking, and research [20]. A scoping review investigating the effects of meditation on loneliness was conducted based on Arksey and O’Malley’s [21] five-stage scoping review framework. The five steps are as follows: (1) identifying the research question, (2) identifying relevant studies, (3) selecting the studies, (4) charting the data, and (5) collating, summarizing, and reporting the results.

### Step 1: identifying the research question

The purpose of the present scoping review was to identify the quantity and type of studies investigating the effects of meditation on loneliness. Study eligibility was based on a Population, Intervention, Comparison and Outcomes (PICO) framework. Eligible *populations* included adults aged 18 years and older experiencing loneliness. With respect to *interventions*, meditation and other mindfulness related practices, such as mindfulness-based stress reduction (MBSR) were the focus of this study and the basis for eligibility; we excluded any therapies with no meditation-specific component (i.e. yoga as a form of exercise). There were no *comparisons. Outcomes* included a summarization and thematic analysis of findings across all eligible articles.

### Step 2: finding relevant studies

Following preliminary searches of the literature, search strategies were developed for and conducted on MEDLINE, EMBASE, AMED, and CINAHL databases. The National Centre for Complementary and Integrative Health (NCCIH), and the American Psychological Association (APA) websites were also searched for eligible primary research articles and scoping or systematic reviews evaluating outcomes on use of meditation in individuals facing loneliness. Reference lists of scoping and systematic reviews were also reviewed for other potentially eligible primary research articles not captured by our search strategies. The search, designed by GKS and JYN, included literature published from database inception up and including the week of April 22, 2020. Terms searched included “breathing exercise(s)”, “loneliness”, “meditation”, “mind–body therap(ies)”, “mindfulness”, “patient isolation”, “relaxation therap(ies)”, “social distance”, and “social isolation”. These terms were identified following a review of indexed headings and keywords of articles found in our preliminary searches. A sample search strategy is provided in Table 1.

**Table 1:** MEDLINE search strategy for primary studies examining the effects of meditation on loneliness executed April 28, 2020

Database: Ovid MEDLINE(R) and Epub Ahead of Print, In-Process and Other Non-Indexed Citations, Daily and Versions(R) <1946 to April 22, 2020>
*Search strategy*
1 Loneliness.mp. or Loneliness/ (7054)
2 Social distance.mp. or Social Distance/ (3387)
3 Social isolation.mp. or Social Isolation/ (17,414)
4 Patient isolation.mp. or Patient Isolation/ (4045)
5 Or/1–4 (30,399)
6 Breathing Exercises/ or breathing exercise*.mp. (3974)
7 Meditation.mp. or Meditation/ (5937)
8 Mindfulness.mp. or Mindfulness/ (7708)
9 Mind–Body Therapies/ or mind–body therap*.mp. (1289)
10 Relaxation Therapy/ or relaxation therap*.mp. (6671)
11 Or/6–10 (21,834)
12 5 and 11 (94)
13 Limit 12 to english language (91)

### Step 3: selecting the studies

Only primary research articles evaluating the effect of meditation on loneliness were included for the purpose of this scoping review. Articles were excluded at this stage if they did not make reference to our research objective of alleviating loneliness using meditation. If studies identified or measured outcomes resulting from the effects of meditation on loneliness, they met our inclusion criteria; this was true regardless of whether these aforementioned outcomes were designated by the study as primary or secondary. Publications in the form of protocols, abstracts, letters, editorials, case reports or case series were not eligible. We also restricted our eligibility criteria to articles published in the English language, those that involved adult populations (aged 18 +), and that were either available publicly or could be ordered through the McMaster University library system. All authors (GKS, SBH, ZT, and JYN) initially pilot-screened a subset of the titles and abstracts independently and then met to discuss and resolve discrepancies. Following deduplication, all search results were independently screened in triplicate (GKS, SBH, and ZT). All four authors then met, and any discrepancies were resolved; in the case that a consensus could not be reached, a majority vote was held (between GKS, SBH, and ZT) following discussion with the supervising author (JYN).

### Step 4: charting the data

The articles that met the inclusion criteria were critically reviewed using Arksey and O’Malley’s descriptive-analytical narrative method [21]. For each eligible article that was included, the following data was then extracted and charted: article title, author(s), year of publication, study country, study setting, study design, population type and sample size, definition of loneliness, type of meditation used, duration of meditation, the occurrence of a follow-up (and duration), primary and secondary outcomes and how they were measured, main findings, challenges encountered, and conclusion. GKS and JYN developed the data extraction forms. GKS, SBH, and ZT completed data extraction for a subset of the eligible articles and then met to discuss and resolve any discrepancies. This was followed by the three authors (GKS, SBH, and ZT) completing a full data extraction of all the eligible articles independently and meeting to discuss and resolve any discrepancies, with JYN resolving discrepancies within both the pilot and final data extraction sets when a consensus was unable to be reached.

### Step 5: collating, summarizing, and reporting the results

Charted data was summarized in the format of tables, followed by descriptive data being analyzed using thematic analysis. GKS reviewed the entire set of data, while SBH and ZT reviewed subsets of the data. JYN and GKS identified codes relative to the findings, organized codes into thematic groups, and presented a narrative relating to the research question, as well as highlighted knowledge gaps in the currently existing literature. All four authors then met to discuss and resolve discrepancies.

## Results

### Search results

**Fig. 1:**
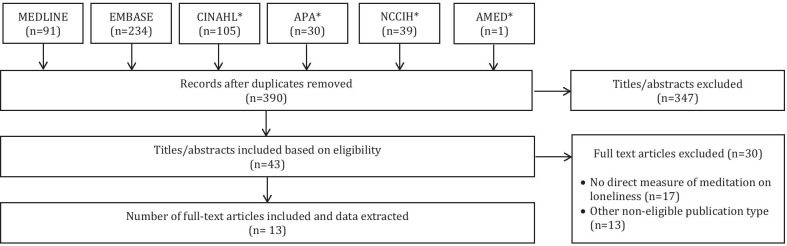
PRISMA diagram. *CINAHL* Cumulative Index to Nursing and Allied Health Literature, *APA* American Psychological Association, *NCCIH* National Center for Complementary and Integrative Health, *AMED* Allied and Complementary Medicine

Searches identified a total of 500 items, of which 390 were unique, and 347 titles/abstracts were eliminated, leaving 43 full-text articles to be considered. Of those, 30 were not eligible, because they did not evaluate loneliness using a meditation intervention (n = 17) or were a non-eligible publication type (n = 13), resulting in a total of 13 eligible articles [9, 22–33]. A PRISMA diagram depicting this process is shown in Fig. 1.

### Eligible article characteristics

Thirteen studies met the inclusion criteria and were published between 2012 and 2020 and conducted across 10 countries [9, 22–33]. These articles originated from the United States (n = 7); Australia (n = 1); Germany (n = 1); India, Nepal, Myanmar,  and Sri Lanka (n = 1); Iran (n = 1); South Korea (n = 1); and Spain (n = 1). Of the 13 articles, 8 were randomized controlled trials (RCTs), 2 were feasibility studies with an RCT design, 1 was a feasibility and acceptability study, 1 was a pilot study looking at feasibility, and 1 was a quasi-experimental study. Six of the 13 studies (46%) used meditation in combination with other interventions, such as yoga. The remaining 7 studies (54%) used meditation as the only intervention, the types of which included cognitively based compassion training, brain education-based meditation, tai chi meditation, and mindfulness-based stress reduction training. Ten out of 13 studies (77%) looked at loneliness in some aspect as a primary outcome, while 3 out of 13 studies (23%) looked at loneliness as a secondary outcome. General characteristics of all eligible studies are found in Table 2 and details specific to findings and outcomes are found in Table 3. Additional demographic characteristics of all eligible studies are provided in Table 4.

**Table 2:** General characteristics of eligible studies

Author and year	Title	Country	Study setting	study design	Population and sample size	Loneliness definition	Meditation type	Duration of meditation	Was there a follow-up	If yes, duration of follow-up
Brooker et al. 2020 [22]	A feasibility and acceptability study of an adaptation of the Mindful Self-Compassion program for adult cancer patients	Australia	Education and research institute building	Feasibility and acceptability study	Patients who had received a diagnosis of non-advanced cancer in the previous 6 months—5 years, who resided within 60 km of the intervention venue, were fluent in English, anticipated that they could attend at least four of the Mindful Self-Compassion (MSC) group sessions, and were able to provide written informed consent; ss: n = 30 at the beginning of the study, n = 27 at conclusion	No definition provided	Written reflective practices and guided meditations supported by audio-recordings made available by the program developers + daily homework component of approximately 20 min of both informal and formal self-compassion and mindfulness practices (i.e. core meditation, loving-kindness, opening meditation, meditation: compassion for self and others). Group-based and instructor-led MSC program adapted to cancer patients, face-to-face	At least 4 sessions of 105 min, delivered weekly. Therefore 4 weeks. Minimum attendance required of 4 sessions out of 8, so could have gone up to 8 weeks	No	Not applicable
Rodriguez-Romero et al. 2020 [23]	Intervention to reduce perceived loneliness in community-dwelling older people	Spain	Urban primary healthcare center	Randomized clinical trial without blind evaluation	Community dwellers with moderate to severe perceived loneliness (according to UCLA Scale) and moderate autonomy or dependence (according to Barthel Index); ss: n = 55	Looked at perceived loneliness. "Loneliness, a subjective phenomenon derived from the discrepancy between the level of social contact achieved and that desired, is described as a painful experience accompanied by negative feelings."	Group-based mindfulness session/workshop to reduce or minimize stress (face-to-face)	18 sessions over 6 months	No	Not applicable
Lee et al. 2019 [24]	Brain education-based meditation for patients with hypertension and/or type 2 diabetes	South Korea	Public health center	Pilot non-blinded randomized control trial (RCT)	48 patients with hypertension and/or type 2 diabetes; 24 to Brain Education-based Meditation (BEM) and 24 to health education—at completion there were 14 in control and 21 in BEM; ss: total of 35 post-intervention	No definition provided	Instructor-led BEM, which is a technique known to change brain structure, psychology, and physiology of healthy adult participants. Group classes	8 weeks	No	Not applicable
Lindsay et al. 2019 [25]	Mindfulness training reduces loneliness and increases social contact in a randomized controlled trial	United States	Smart-phone delivered intervention (remote, at-home study activities)	Three-arm randomized controlled dismantling trial	153 healthy but stressed English-speaking adults who owned smartphones	No definition provided	Smartphone based lessons consisting of training in either: monitoring and acceptance, monitoring only, or active control training. Lessons consisted on 20-min of audio in addition to brief homework practice	2 weeks	No	Not applicable
Pandya 2019 [26]	Meditation program mitigates loneliness and promotes wellbeing, life satisfaction and contentment among retired older adults: A two-year follow-up study in four South Asian cities	India, Nepal, Myanamar, Sri Lanka	Voluntary agencies located in each of the 4 South Asian cities	RCT	Retired older adult members (retired for 2–5 years at start of study); ss: Post-test, 166 remained with intervention and 157 with control (n = 323)	Loneliness is the perceived absence of positive social relationships and intimate relationships, varies based on personal and contextual determinants and is a negative emotional experience. Loneliness is an outcome of the subjective, cognitive evaluation of there being a mismatch between the quality and quantity of existing relationships on the one hand, and relationship standards on the other	Customized meditation program (instructor-led and in groups), the key features of which were: (i) postures interspersed with relaxation, (ii) slowness in movements and, (iii) inner watchful awareness. This customized meditation program comprised a 45-min guided lesson conducted once a week by instructors and the same was to be practiced at home by the participants once a week prior to the next class (individually)	2 years	No	Not applicable
Mascaro et al. 2018 [27]	Meditation buffers medical student compassion from the deleterious effects of depression	United States	Medical school campus	Feasibility study with randomized single-blind wait-list controlled trial	11 in wait-list and 21 in CBCT = 32 completed study (59 initially enrolled)	No definition provided but it is specified to be experienced by medical students experiencing burnout	CBCT (consisting of in-person instructor-led group classes in addition to at-home practice)	10 weeks	No	Not applicable
Kok et al. 2017 [28]	Effects of contemplative dyads on engagement and perceived social connectedness over 9 months of mental training	Germany	Not specified (both online and in-person interventions took place)	RCT	242 healthy participants after excluding participants with not applicable data	A perceived lack of social connectedness	Secularized classical solitary meditation training modules made up of: breathing meditation and body scan (the presence module), loving-kindness meditation and affect dyad (the affect module), and observing-thoughts meditation and perspective dyad (the perspective module) on website and phone app—consisting of an in-person 3-day retreat, online individual modules and guided meditations, and online 2-person dyad training	9-month open-label efficacy trial of three, 3-month secularized mental training modules	No	Not applicable
Tkatch et al. 2017 [29]	A pilot online mindfulness intervention to decrease caregiver burden and improve psychological well-being	United States	2 local community centers	Pilot study examining feasibility of intervention	Community-dwelling older adult caregivers (n = 40)	No definition provided	Online mindfulness intervention; combination of education, mindfulness meditation, and self-care, which primarily focused on self-compassion. Eight modules were delivered twice weekly online, utilizing phone and web-interface to an online learning platform that contained session materials, downloadable brief meditation practices, access to short learning videos, and other support tools. 3 of the modules were also delivered in-person to provide alternative touch points if participants were able to attend and preferred this method	8 weeks	No	Not applicable
Dodds et al. 2015 [30]	Feasibility of Cognitively-Based Compassion Training (CBCT) for breast cancer survivors: a randomized, wait list controlled pilot study	United States	Recruitment from the University of Arizona Comprehensive Cancer Center; location of classes not specified. At home and in-class sessions occurred (unclear if class is in-person or online)	Feasibility study with a randomized wait list control trial design	Women with a history of breast cancer treated with adjuvant systemic chemotherapy within the past 10 years (with no current chemotherapy other than prophylactic use of a selective estrogen-receptor modulator); ss: n = 33	No definition provided	CBCT with training in concentrative and mindfulness practices consisting of in-person instructor-led group classes in addition to at-home practice consisting of guided meditations	8 consecutive weeks of classes followed by a "booster" class at week 12 (4 weeks later)	Yes	1 month
Samhkaniyan et al. 2015 [31]	The effectiveness of mindfulness-based cognitive therapy on quality of life and loneliness of women with HIV	Iran	Not specified	Quasi-experimental model with pretest–posttest and check team	Women who were human immunodeficiency virus (HIV) positive (n = 24)—12 in control and 12 in Mindfulness-Based Cognitive Therapy (MBCT)	The difference between current and ideal situations in achieving communication with others and society	MBCT	8 weeks	No	Not applicable
Black et al. 2014 [32]	Tai chi meditation effects on nuclear factor-kB signaling in lonely older adults: A randomized controlled trial	United States	Not specified	RCT	Lonely older adults (naïve to tai chi and scored 40 + on UCLA Loneliness scale); ss: 26 randomized (split evenly), 22 completed postintervention visit—10 in Tai Chi Chih (TCC), 12 in stress and health education (SHE)	No definition provided	Group-based tai chi meditation—meditation technique, TCC consisted of 20 guided meditative movements under the instruction of a certified teacher	12 weeks group-based program delivered weekly in 2-h sessions	No	Not applicable
Creswell et al. 2012 [9]	Mindfulness-Based Stress Reduction training reduces loneliness and pro-inflammatory gene expression in older adults: A small randomized controlled trial	United States	Not specified	RCT	40 healthy older adults interested in learning mindfulness meditation techniques, recruited via newspaper advertisement	A state of social distress that arises when there is a discrepancy between one’s desired and actual social relationships	Mindfulness-Based Stress Reduction (MBSR) program, which is a mindfulness meditation intervention. During each group session, an instructor led participants in guided mindfulness meditation exercises, mindful yoga and stretching, and group discussions with the intent to foster mindful awareness of one’s moment-to-moment experience. This is done in addition to a day-long retreat and at-home mindfulness practice	8 weeks	No	Not applicable
Jazaieri et al. 2012 [33]	A randomized trial of MSBR versus aerobic exercise for social anxiety disorder	United States	Eight healthcare settings throughout the San Francisco Bay Area (specifics about the types of healthcare settings are not available)	RCT	Adults who met DSM-IV criteria for generalized social anxiety disorder (n = 56 in intervention group), n = 48 healthy adults in control group	No definition provided	Standard MBSR program, which comprises eight, weekly 2.5-h group classes, a 1-day meditation retreat, and daily home practice. Participants were trained in formal meditation practice (i.e., breath focus, body scan, open monitoring), brief informal practice, and Hatha yoga. Forms to monitor daily meditation and yoga practice were collected each week (in-person)	8 weeks	Yes	3 months

*BEM* brain education-based meditation, *CBCT* cognitively based compassion training, *HIV* human immunodeficiency virus, *MBCT* mindfulness-based cognitive therapy, *MBSR* mindfulness-based stress reduction training, *MSC* mindful self-compassion, *RCT* randomized control trial, *SHE* stress and health education, *SS* sample size, *TCC* Tai Chi Chih

**Table 3:** Outcomes and findings of eligible studies

Author and year	Primary Outcomes	How Primary Outcomes Were Measured	Secondary Outcomes	How Secondary Outcomes Were Measured	Main Findings	Challenges Encountered	Conclusion
Brooker et al. 2020 [22]	To examine the feasibility and acceptability of an adaptation of the MSC program among adult cancer patients	Acceptability (to clinicians) determined by proportion of clinicians approached who agreed to recruit patients to the study Feasibility of the mail-out recruitment method was operationalized by the percentage of these clinicians who facilitated a mail-out Acceptability (to invited patients) determined by the percentage of potential participants who consented to the program, with a target of 10–20% Acceptability (among those who commenced the program) assessed by retention rates, with 70% target of at least four group session attendance -Acceptability also measured using a MSC program evaluation form, adapted for cancer context, included in questionnaire after final group session	To examine pre–post-program changes in psychosocial wellbeing: symptoms of depression and stress, fear of cancer recurrence, loneliness, body image, self-compassion, mindfulness	Depression and stress symptoms measured by 21-item Depression Anxiety Stress Scales (DASS-21) Fear of cancer recurrence or progression measured by 9-item Fear of Cancer Recurrence Inventory-Short Form (FCRI-SF) Loneliness measured by 20-item UCLA Loneliness Scale Version 3 Body image assessed using 10-item Body Appreciation Scale (BAS) Version 2 Mindfulness measured using Cognitive and Affective Mindfulness Scale-Revised Positive and negative facets of the three self-compassion components measured using 26-item Self-Compassion Scale (SCS)	Positive: Feasibility and acceptability: 13 of 17 (76%) of approached clinicians agreed to recruit patients; 19% of contacted patients consented to the program—in total, 32 participants consented to the program, with 30 commencing it and 27 completing it Results of Intervention: Significant decrease in loneliness (medium to large effect), depression (large effect), stress (medium to large effect), and fear of cancer recurrence (medium to large effect) in intervention group. Significant increase in mindfulness (large effect) Nonsignificant increase in body appreciation (small to medium effect) and self-compassion (medium to large effect)	Study Design: Study did not include a control arm and had a small sample size, making it difficult to attribute pre- to post-intervention changes to intervention. Study had a small sample size Program Length: Shortened version of the program was delivered (14 vs 20 h) which may have accounted for smaller effect sizes Recruitment Strategy: Patients were invited to participate by treating clinicians, which may have elevated perceived acceptability compared to other channels	The adaptation of an 8-week mindfulness and self-compassion program for patients with non-advanced cancer diagnosis is feasible and acceptable. Preliminary findings indicate that the intervention has significant increases in psychosocial well-being and loneliness. However, additional studies with a control group must be undertaken to validate results
Rodriguez-Romero et al. 2020 [23]	Loneliness, social support, depression, and quality of life (physical and mental)	Perceived loneliness assessed by the UCLA scale Degree of autonomy measured by the Barthel Index Degree of cognitive impairment assessed by the Pfeiffer test Depressive symptoms using the Yesavage abbreviated questionnaire Perceived social support evaluated by the Duke-UNC-11 Functional Social Support Questionnaire Perceived quality of life measured by the 12-Item Short Form Health Survey (SF-12) Group sessions assessed by intervention group participants using a satisfaction survey at the end of the programme Sessional attendance collected using a weekly attendance record	Not available	Not applicable	Positive: Improvements in all measures except for the SF-12 (quality of life) physical component -Significant improvement in degree of loneliness within the intervention group, with no perceived loneliness shown at 6 months by 14 (48.3%) participants Nonsignificant increase in control group patients with severe loneliness at 6 months (7.7% preintervention vs. 19.2% postintervention) Mean improvement in degree of loneliness was 8.63 points (95% CI 1.97–15.30) higher in the intervention group in comparison to the control group (p = 0.012)	Small sample size: Of the 80 referred patients, 25 were excluded since 6 were unavailable throughout the study, 6 didn't experience loneliness, 6 didn't attend sessions due to caring for a relative 24 h a day, 6 didn't have enough time, and 1 refused to participate. It is hard to recruit lonely people, as seen through the small sample size, which suggests that the community intervention reached primarily people with moderate loneliness; not those with severe loneliness, who might benefit from a more personalised approach Study design: Did not allow for measurement of the intervention benefits over time due to a lack of follow-up once the intervention was complete	After participating in a community intervention promoting socialisation, almost half of the lonely older persons stopped feeling lonely and their health status improved. During this same period of time, controls with a similar baseline showed no change in their perception of loneliness or other health variables. These results are similar to the findings in other studies, however, a smaller decrease in loneliness is seen in studies of individual interventions
Lee et al. 2019 [24]	Whether BEM affects the conditions of patients with hypertension and/or type 2 diabetes compared with health education classes (Mental/physical health and wellbeing: relaxation, focus, happiness, confidence, reduction in anger, loneliness)	Blood collection, followed by measurement of serum glutamic-oxaloacetic transaminase, serum glutamic pyruvic transaminase, γ-glutamyl transferase, creatinine, high-density lipoprotein cholesterol, and low-density lipoprotein (LDL) cholesterol Ribonucleic acid (RNA) extraction, complementary deoxyribonucleic acid synthesis and reverse transcription polymerase chain reaction of inflammatory genes Self-reported questionnaires of mental and physical health of 20 items with a 5-point Likert-type scale for each response	Not available	Not applicable	Positive: Intervention (BEM) group showed significant decreases in LDL cholesterol post-intervention, while control group did not. Intervention group also had significant reductions in expression of inflammatory genes Mental and Physical Health: Post-intervention self-report scores for the intervention group showcase significant increases in focus, confidence, relaxation and happiness, along with significant decreases in fatigue, anger and loneliness There were no important adverse events or side-effects by BEM intervention	Study Design: Relatively small sample size (follow-up with a larger sample needed to validate findings). Intervention (BEM) included both static and dynamic elements making it difficult to know which elements contributed to which results. More measurements required such as hemoglobin A1c and body mass index (BMI) Other Factors Responsible for Effects: Medication types were not controlled for, therefore some of the medication taken by participants may have impacted results	The results of the pilot-randomized controlled trail showcase that a BEM intervention reduces LDL cholesterol and inflammatory gene expression, in addition to improving mental and physical health in patients with type 2 diabetes/hypertension, compared to health education. Since both type 2 diabetes and hypertension are chronic conditions, positive effects from non-invasive interventions such as BEM are significant for long-term complementary care
Lindsay et al. 2019 [25]	Assessing loneliness and social isolation/social interaction in participants’ natural environments, as well as global retrospective measures of loneliness and social support. Social Processes (subjective perception of loneliness, objective number of social interaction and partners)	Social processes measured by Ecological Momentary Assessment, carried out 4 quasi-random times a day, and End-of-Day Surveys Retrospective loneliness measured by UCLA Loneliness Scale Retrospective social isolation measured by Social Network Index Retrospective social support measured by Interpersonal Support Evaluation List Reactions to social interactions were measured using a subset of Ecological Momentary Survey	Not available	Not applicable	Positive Outcome for Some Interventions: Patients undergoing monitor and acceptance mindfulness meditation training showed significant decreases in loneliness and significant increases in social interactions pre- to post-intervention. In contrast, patients in the monitor only or control group did not showcase any significant differences in loneliness and social interactions pre- to post-intervention Overall, monitor and acceptance training reduced daily-life loneliness by 22%, and increased social interactions by 2 more interactions per day and one more person per day compared to monitor only or control training	Study Design: Sampling (Recruited a sample of stressed community adults rather than specifically targeting socially isolated individuals) and follow-up (no follow-up included in study) Further research is needed to test whether smartphone-based mindfulness meditation training can reduce loneliness within lonely population Further research is also needed to identify whether mindfulness training helps strengthen current relationships or aids in the formation of new relationships	This 2-week trial provides evidence that individually delivered smartphone-based mindfulness training can reduce loneliness and increase social contact in daily life. Importantly, the differences between intervention groups showcased the importance of developing an accepting attitude towards present experiences in order to decrease loneliness and improve social contact
Pandya 2019 [26]	Loneliness, Well-Being, Life Satisfaction, Contentment with Life	6-Item de Jong Gierveld Loneliness Scale Warwick-Edinburgh Mental Wellbeing Scale 5-Item Satisfaction with Life Scale (SWLS) Contentment with Life Assessment Scale	Not available	Not applicable	Positive: Pre-Intervention: There were no significant differences in psychosocial measures within the intervention and control group at baseline Post-Intervention: No significant increases in measures for control group. In contrast, the intervention group showed a significant decrease in loneliness, significant increase in mental well-being, satisfaction with life, and contentment with life. Furthermore, all post-intervention outcome measures for the invention group were higher than for the control group	Study Design: Data was only gathered at 2 specific time points, which does not account for interim changes. No qualitative data was collected to study perceptions of intervention. Self-practice was an important predictor for success, but variances in conditions for self-practice were not tracked or controlled Sampling and Study Population: Study population had a lot of heterogeneity, mostly compromising middle-class married Hindu men	A customized meditation program can significantly alleviate loneliness in older adults. It may be customized and refined for different demographics such as women, upper class individuals, single vs married, living alone vs cohabiting with family, Buddhists, and those with congenital chronic ailments. Loneliness-mitigation is through building older participants’ inner resources rather than networks and social skills. Program impact is dependent on regular attendance and home practice
Mascaro et al. 2018 [27]	(1) investigate the feasibility of CBCT for second-year medical students, and (2) test whether CBCT decreases depression, enhances compassion, and improves daily functioning in medical students. (3) who benefited most from compassion meditation by testing the hypothesis that CBCT would have the greatest impact on compassion among those suffering from depression, stress, and anxiety	Digital or paper self-report questionnaires pre and post intervention Interpersonal health measured using Compassionate Love for Humanity Scale UCLA Loneliness Scale (R-UCLA) Sleep quality assessed using Pittsburgh Sleep Scale Quantity and frequency of substance use such as tobacco, marijuana, alcohol, or prescription drugs, measured using Substance Use Inventory Negative emotions measured using DASS Frequency of physical and aerobic exercise over the past month measured by asking two questions as part of assessment	Not available	Not applicable	Positive: Individuals randomized to CBCT intervention reported significant decrease in depression and loneliness, along with an increase in compassion and less exercise post-intervention. There were no significant changes in the wait-list control group post-intervention Furthermore, there was a significant main effect of time in the CBCT intervention group for increases in compassionate love and sleep, and decreases in loneliness, depression and exercise	Sample size: Small Other non-specific factors may have influenced outcomes Study does not provide significant insight into the complex nature of socio-cognitive changes that may result from CBCT or how they may alter physician competence or patient outcomes	The study's findings indicate that CBCT may be beneficial for enhancing compassion and reducing depression and loneliness for medical students. Further studies are needed to study mechanisms of the CBCT effect, along with its long-term effects and impacts of patient outcomes
Kok et al. 2017 [28]	Self-disclosure and social closeness Engagement Measures (compliance, liking, motivation to practice) Outcome Measures (Closeness of dyadic partner, self-disclosure)	Compliance measured through ranked scale reporting of motivation and liking Closeness measured with Inclusion of Other in the Self Scale Self-disclosure using Ranked Scale for Self-Disclosure Rating	Valence and arousal	Valence and arousal assessed before and after all sessions, assessing affect with scales using ranges of 0 to 8	Positive: All participants practiced breathing meditation and body scans (presence modules), followed by dyadic practice (affect module and perspective module). Compliance was similar across all modules, while motivation was higher for the meditation modules Social closeness showed significant improvements during a session for the affect dyads and significant increase over time for the affect dyad. Self-disclosure increased over time for the affect dyad and the perspective dyad, with the perspective dyad showcasing a greater rate of increase (both significant results)	Study Design: Trial of contemplative dyads was embedded with a larger mental training study; therefore, the effects of contemplative dyads on social closeness and self-disclosure needs to be studied independently Study Population: Included only health adults; further research with populations at greater risk of suffering from loneliness, such as older adults or adults with maladaptive social cognitions, is needed	In this trial, 2 types of contemplative dyadic exercises practiced over 6 months increased social closeness and self-disclosure among participants. Provides evidence for new type of intervention targeting social connection in individuals who may suffer from loneliness
Tkatch et al. 2017 [29]	To determine if this intervention could be a feasible approach for this population (community-dwelling older adult caregivers). Feasibility was initially assessed by the ability to attract participants and sustain engagement in the intervention	Attendance of the weekly (online and in-person) modules was recorded	Impact of the intervention on caregiver burden, quality of life (mental and physical well-being), and psychological well-being measures, including stress, loneliness, anxiety, and social support	Baseline and post-treatment surveys measured the impact of the intervention, such as caregiver burden, quality of life, psychological well-being measures such as loneliness, stress, anxiety, social support Caregiver burden measured by Zarit Short Burden Interview 12-item Veteran’s Rand 4-item Perceived Stress Scale 7-item Generalized Anxiety Disorder Test 3-item UCLA Loneliness Scale 12-item Interpersonal Support Evaluation List	Positive: Post-intervention, participants had significant decreases in caregiver burden, stress, loneliness, and anxiety, and significant increases in mental health Higher levels of session attendance was significantly associated with positive changes in perceived social support and the mental component score, along with significant decreases in stress and anxiety	Study Design: No control group and small sample size Study Population: Participants were recruited from an existing caregiver support group and therefore, might have a greater need for support and a willingness to participate, decreasing generalizability to all caregivers	This pilot study provides evidence that online mindfulness meditation programs have the potential to significantly reduce caregiver burden, along with improving mental health for older caregivers. Future studies could expand on results by testing with a larger sample size and longitudinal cohorts or targeting caregivers of older adults with special needs or young children
Dodds et al. 2015 [30]	The feasibility of a meditation-based program (CBCT) with breast cancer survivors treated with systemic adjuvant chemotherapy within past 10 years	Recruitment rate Screening and enrollment rate Class attendance Adherence Retention Participant satisfaction and interest	The impact of CBCT on behavioural endpoints (Perceptions of Loneliness and Social Connectedness, Perceived Stress, Depression, Psychological Distress and Functioning Impairments linked to fear of cancer recurrence, Intrusive Thoughts, Avoidance and Hyperarousal, Pain and Vitality, Global Attention, Awareness, Present Focus and Acceptance, Gratitude, Satisfaction) Diurnal rhythm of cortisol (a stress-related endocrine biomarker) Impact of home practice on outcomes (Adherence to CBCT Protocol)	Salivary cortisol collection through at-home collection kits 4-item Perceived Stress Scale (PSS-4) Brief Center for Epidemiologic Studies—Depression questionnaire Five subscales of the FCRI Impact of Events Scale—Revised R-UCLA Version 3 Medical Outcomes Study SF-12 Cognitive and Affective Mindfulness Scale—Revised Gratitude Questionnaire—6, participant satisfaction measured by two items	Positive: Positive but non-significant findings in relation to loneliness Feasibility and Acceptance: Attendance and participant satisfaction met the pre-defined criteria, while retention, home meditation practice days and recruitment rate were slightly under goals Behavioural and Psychosocial Outcomes: Significant decreases in depressive symptoms, functional impairments from fear of cancer recurrence, avoidance were observed pre- to post-intervention, in addition to a significant increase in mindful presence Loneliness: Nonsignificant decrease in loneliness post-intervention and at 1-month follow-up Cortisol: No effect of CBCT was observed on any measure of cortisol (including diurnal cortisol rhythm)	Recruitment Rate: Lower than planned, with a bias towards participants with higher. socioeconomic status Potential Bias: Potential positive impact of self-reporting problems commonly experienced during survivorship Data Collection: 3 Participants did not return practice log data at follow-up period Effects of Intervention: Possible ceiling effect with participants engaging in at-home meditation practice to the point where a positive correlation is less likely to be seen	Within the limits of a pilot feasibility study, results suggest that CBCT is a feasible and highly satisfactory intervention potentially beneficial to the psychological well-being of breast cancer survivors. However, more comprehensive trials are needed to provide systematic evidence
Samhkaniyan et al. 2015 [31]	The performance of mindfulness according to the cognitive approach on the quality of life and loneliness of women with HIV	Quality of life survey from WHO and revised UCLA to both groups pre and post intervention	Not available	Not applicable	Positive: MBCT resulted in increases in mean quality of life and non-significant decreases in loneliness (when comparing pre- and post-intervention scores within the intervention group only, and between intervention and control group)	Sample Size: Small sample size, which could minimize generalizability and significance of results No similar studies in Iran to validate/compare conclusions to	This study provides evidence that group based MBCT is effective in reducing loneliness and increasing the quality of life in women with HIV
Black et al. 2014 [32]	Affect of TCC on psychologic stress and NF-κB levels in lonely older adults, as compared to those who receive a stress and health education (SHE) intervention	14-Item PSS Blood sample collection and measurement of serum nuclear factor (NF)-kB	Not available	Not applicable	Unclear/Positive Findings: In the health education control group, psychological stress levels were unchanged, while nuclear levels of activated NF-κB significantly increase post-intervention In the Tai Chi intervention group, levels of psychological stress significantly decreased, while NF-κB levels remained unchanged post-intervention	Study Population: Small sample size consisting predominantly of women, limiting generalizability of results Lack of Measurements: NF-κB levels were only measured in peripheral blood mononuclear cells (PBMCs); therefore, observed changes may be due to changes in lymphocyte subset distribution	TCC significantly reduced levels of psychological stress compared to the control group, while attenuating the rise of NF-κB activation in lonely older adults
Creswell et al. 2012 [9]	Effects of MBSR on loneliness and loneliness-related inflammatory genes such as NF-kB	Perceived loneliness assessed by the UCLA scale, revised (UCLA-R) at baseline and post-treatment Blood sample collection and RNA extracted from PBMCs Mindfulness measured using 39-item Kentucky Inventory of Mindfulness Skills Bioinformatic indications of increased expression of pro-inflammatory genes like NF-kB transcription factor, monocyte-mediated gene expression (while controlling for sex, age, ethnicity, and BMI) Different white blood cell subtypes of gene expression changes determined using transcript origin analysis C-reactive protein and interleukin-6 levels measured in EDTA samples by high sensitivity ELISA	Effects of MSBR on self-reported sleep quality and exercise	-Sleep quality measured using Pittsburgh Sleep Quality Index measure -Self-reported exercise	Positive: MBSR intervention group showcase a significant decrease in loneliness post-intervention, compared to a small decrease in the wait-list control group. Similarly, after controlling for baseline loneliness, MBSR intervention group had significantly lower loneliness scores post-intervention than control MBSR intervention group also had significant reductions in activity of NF-κB target genes post-intervention compared to control group No significant effect of intervention on sleep quality was observed	Study Design: Wait list control group instead of an active comparator arm Intervention Design: Mindfulness meditation was taught in groups instead of individually. Teaching it individually may have shown an increased effect	The study provides evidence that an 8-week MBSR intervention reduces perceptions of loneliness in older adults. Evidence also supports that loneliness is associated with increased activity of NF-κB target genes and that MBSR can significantly downregulate this gene expression in parallel to loneliness reduction
Jazaieri et al. 2012 [33]	Many factors (i.e. clinical symptoms and subjective well-being) measured such as social interactions, loneliness, depressive symptoms, social anxiety, psychological stress, self-esteem, life satisfaction, self-compassion	Liebowitz Social Anxiety Scale-Self-Report Social Interaction Anxiety Scale Straightforward Scale Beck Depression Inventory-II PSS-4 Rosenberg Self-Esteem Scale SWLS SCS, UCLA-8 Loneliness Scale	Not available	Not applicable	Positive: Both MBSR and aerobic exercise (AE) were linked to a reduction in social anxiety and depression, as well as an increase in subjective well-being. Overall, no significant differences in these measures were seen between MBSR and AE Participants in both the randomized controlled trial and the untreated social anxiety disorder (SAD) group showed improvements in their measures of clinical symptoms and well-being	Study Design: The respective group experiences and time entailed in each interaction may be the cause for the apparent differences between the intervention groups Sampling: Study required participants to voluntarily contact researchers to partake in the study, which may have resulted in a study population with less severe social anxiety	Non-traditional interventions such as MBSR and AE produce modest clinically significant changes in social anxiety, depression, and subjective well-being for patients with SAD. However, these changes were not at the same level as what has been found in previous studies with traditional treatments

*AE* aerobic exercise, *BAS* Body Appreciation Scale, *BEM* brain education-based meditation, *BMI* body mass index, *CBCT* cognitively based compassion training, *DASS* Depression Anxiety Stress Scales, *FCRI* Fear of Cancer Recurrence Inventory, *HIV* human immunodeficiency virus, *LDL* low-density lipoprotein, *MBCT* mindfulness-based cognitive therapy, *MBSR* mindfulness-based stress reduction training, *MSC* mindful self-compassion, *NF* nuclear factor, *PBMC *peripheral blood mononuclear cell, *PSS-4* 4-item Perceived Stress Scale, *RNA* ribonucleic acid, *R-UCLA* Revised UCLA, *SAD* social anxiety disorder; *SCS* Self-Compassion Scale, *SF-12*12-Item Short Form Health Survey, *SHE* stress and health education, *SWLS* Satisfaction with Life Scale, *TCC* Tai Chi Chih

**Table 4:** Demographic characteristics of eligible studies

Author and year	Age	Ethnicity	Socioeconomic status	Education
Brooker et al. 2020 [22]	38–85	Australian country of birth (n = 23) Other (n = 4)	N/A	Primary (n = 1) Trade/vocational (n = 7) Undergraduate tertiary (n = 6) Postgraduate tertiary (n = 9)
Rodriguez-Romero et al. 2020 [23]	> = 65 years	Hispanic/Spanish	Income € < 750/month (n = 1) €750–1000/month (n = 22) €1000–1500/month (n = 32)	Primary (n = 48) Secondary (n = 6) University (n = 1)
Lee et al. 2019 [24]	57–87 years	East Asian (Korean)	N/A	N/A
Lindsay et al. 2019 [25]	18–70 years	Hispanic or Latino (n = 7) Not Hispanic or Latino (n = 146)	N/A	GED (n = 3) High School diploma (n = 20) Technical training (n = 1) Some college (n = 41) Associate degree (n = 10) Bachelor's degree (n = 48) Master's degree (n = 26) MD, PhD, JD, PharmD (n = 4)
Pandya 2019 [26]	62–68	South Asian	Middle class (n = 147) Upper class (n = 42)	College degree (n = 77) Higher qualifications (n = 112)
Mascaro et al. 2018 [27]	22–30 years	N/A	N/A	Second year medical school
Kok et al. 2017 [28]	Adults (mean age 41.15)	German—no other information provided	N/A	N/A
Tkatch et al. 2017 [29]	Adults	N/A	N/A	N/A
Dodds et al. 2015 [30]	> = 18 years	White (n = 11) Not White (n = 1)	< $25,000 (n = 0) $25,000-$49,999 (n = 3) $50,000-$99,999 (n = 6) > $100,000 (n = 3)	High school diploma or less (n = 0) Any college (n = 6) Any graduate school (n = 6)
Samhkaniyan et al. 2015 [31]	20–45 years	Iranian (Persian)	N/A	Minimum pre-high school diploma
Black et al. 2014 [32]	67.1 ± 7.2	Caucasian (65%)	Mean 13.8 ± 4.0	Mean 16.9 years of education ± 2.9
Creswell et al. 2012 [9]	55–85	Caucasian (n = 13) African American (n = 2) Asian American (n = 2) Latino(a) (n = 3) Native American (n = 0) Other (n = 0)	N/A	High school diploma (n = 0) Some college (n = 6) College degree (n = 3) Graduate work (n = 11)
Jazaieri et al. 2012 [33]	Adults (mean 32.87 ± 8.83)	Caucasian (n = 13) Asian (n = 14) Hispanic (n = 3) Multiracial (n = 1)	N/A	Mean 16.40 ± 2.00 years of education

### Summary of eligible article findings

Eighty-five percent of the studies (11 out of 13) identified positive improvements in participants’ feelings of loneliness. Of the two remaining studies, one mentioned the alleviation of loneliness, but only looked primarily at social closeness in individuals experiencing loneliness. The other study found a correlation between loneliness and participants’ nuclear factor (NF)-κB levels, which was the measured outcome; however, the direct effects of meditation for loneliness were unclear.

### Findings from thematic analysis

In total, three main themes emerged from our review and are described below.

#### Positive results across all studies

Upon accounting for all eligible articles, one immediate and striking finding included that all thirteen of the studies reported positive findings for at least one of their respective outcomes [9, 22–33]. Of these studies, 11 out of 13 (85%) studies identified improvements in relation to loneliness [9, 22–27, 29–31, 33]. Of these 11 studies, 7 (64%) studies identified a significant decrease in loneliness within their intervention groups [9, 22–24, 26, 27, 29]. Lindsay et al. also found significant decreases in loneliness, however, only in one of the two intervention groups within the study that had used meditative techniques (monitor and acceptance) [25]. Eighteen percent of the studies (2 out of 11) found positive but insignificant decreases in loneliness [30, 31]. Jazaieri et al. found improvements in clinical symptoms, such as loneliness, in both its RCT and untreated social anxiety disorder group [33]. It is also important to note that loneliness (and its alleviation) was not measured uniformly across the 11 studies. The following measures were used to “quantify” the degree of loneliness: UCLA Loneliness Scale, with variations of the scale used within the studies (n = 8); 6-item de Jong Gierveld Loneliness Scale (DJGLS-6) (n = 1); baseline and post-treatment surveys to measure impacts of intervention (n = 1); and self-reported questionnaire (n = 1).

In the 2 remaining (15%) studies, one measured social closeness in lonely individuals, rather than directly evaluating loneliness [28]. The other study attempted to identify the relationship between NF-κB levels and loneliness [32]. These two studies studied loneliness indirectly, thus the direct effect on loneliness was unclear. Overall, these two studies also reported positive findings, with social closeness showing significant improvements, as measured with the inclusion of “other” in a self-scale [28], and the Tai Chi intervention group experiencing a significant decrease in levels of psychological stress, while NF-κB remained unchanged post-intervention using blood samples to measure the NF-κB levels [32].

#### Relatively small randomized control trials conducted over the last decade

Eight of the 13 (61%) eligible studies were RCTs, 2 out of 13 (15%) were feasibility studies with an RCT design, 1 out of 13 (8%) was a feasibility and acceptability study, 1 out of 13 (8%) was a pilot study looking at feasibility, and 1 out of 13 (8%) was a quasi-experimental study. All of the studies were also published between 2012 and 2020. Of the studies with an RCT design, all contained small sample sizes, ranging from 22 to 323 participants, with the definition of small sample size depending upon the individual objectives of each study [34]. Small sample sizes are a limitation, considering that in order to limit biases in the intervention groups, the sample size must be large enough to provide statistical power to detect a clinically meaningful treatment effect [35]. Two out of 8 of the RCT studies (25%) mentioned explicitly that due to their small sample sizes, future replications of the studies with larger sample sizes would be warranted [24, 32]. Three out of 8 (37.5%) of the RCT studies made no mention of a small sample size limitation, even though their sample sizes were 153, 323, and 48 participants respectively [25, 26, 33]. Considering that these three studies created comparisons between two or more groups of subjects, larger studies are needed in order to distinguish between a real effect and random variation [34]. Kok et al. also did not make any mention of having a small sample size (n = 242, after exclusions), however, this may be due to the fact that this trial was embedded within the context of a larger study regarding mental training for healthy adults [28].

#### Psychosocial factors and age

Forty-six percent (6 out of 13) of the studies were conducted in populations aged 55 and older [9, 23, 24, 26, 29, 32]. As was noted in many of these studies, loneliness and social isolation are more prevalent in elderly populations, which can lead to numerous negative health outcomes [36]. Rodriguez-Romero et al. stated that loneliness can lead to physical, psychological, and social consequences on health within elderly people, a greater risk for cardiovascular disease, depressive symptoms, and a worsened quality of life [23]. Pandya et al. focused on discussing the mental health implications that loneliness has on older adults, stating that the transitional life events this demographic faces (i.e. retirement and bereavement) can trigger loneliness [26]. Black et al. and Creswell et al. similarly outlined that older aged people facing loneliness are at a greater disposition of all-cause mortality and morbidity [32] and have an increased expression of inflammatory genes, which leads to an increased risk of negative health outcomes [9]. Of the six studies focused on older demographics, 33% (2 out of 6) did not provide age-specific information regarding the negative effects of loneliness [24, 29]. The negative outcomes seen in the older demographic is due to the fact that older people are likely to live alone and tend to be less socially engaged as a result, leading to what is referred to as a loneliness epidemic [37]. Rodriguez et al. also emphasized that those aged over 64 years are more likely to face loneliness if they live alone [23]. Two other studies (33%) collected demographic data on study participants who lived alone [26, 29]. Five out of 6 (83%) of these studies found an improvement in loneliness levels through the use of meditation; the remaining study explored the effects of Tai Chi and meditation on NF-κB signalling in lonely older adults, rather than on loneliness directly [32].

#### Demographic information

Fifteen percent (2 out of 13) of the studies did not provide information regarding the ethnicities of the included participants. The articles that included this information provided a range of ethnic participants, with one study including all South Asian participants [26] and another including all Hispanic/Spanish individuals [23]. Since the studies included collectively comprised of participants from different regions around the world, studies included domestic participants that reflected the make-up of their countries. However, of these participants, it is unknown how many of them were immigrants and how many are native to their countries. Those that experience migration often have feelings of loneliness, which would have been a factor of importance to explore [38].

Nine out of 13 (69%) of the articles did not provide socioeconomic information indicating the income and class levels of participants, and 3 out of 13 (23%) provided no indication of the education levels of participants. With respect to socioeconomic status, the studies that provided this information did have a range of participants from various classes, though there was an evident lack of lower-class individuals. Only one study had a single participant who earned less than €750 a month [23], while no other study provided any evidence of including participants that fell into the lower-socioeconomic class category. Given that current research evidence indicates that loneliness is associated with health-risk behaviours in deprived neighbourhoods, thereby indicating associations to socioeconomic statuses, the inclusion of participants from these areas would have provided further insight into the effects of meditation for these individuals [39]. This can similarly be said about the education component since one’s socioeconomic status can directly correlate to their education level [40]. Most of the studies included participants with some sort of schooling, usually having a minimum of a GED or high school diploma. Only 3 of the 10 studies that provided education information included participants without this qualification, with 2 of the studies including participants with only a primary school education [22, 23] and 1 study having a minimum of a pre-high school degree requirement [31]. Overall, within the included studies there was a lack of reporting accounting for the diversity (or lack thereof) comprising  the demographic characteristics of included participants.

## Discussion

The purpose of the present scoping review was to identify the quantity and type of studies investigating the effects of meditation on loneliness. The available quantity of eligible studies on this topic was relatively small, however, of the 13 eligible studies identified, all reported positive findings. This warrants further research to more comprehensively explore and evaluate the benefits of meditation for those facing loneliness. To our knowledge, this is the first systematically-searched review to report on this topic; our findings, therefore, provide both healthcare providers and researchers with a greater awareness of the quantity and type of research studies that have been conducted at the intersection of meditation and loneliness.

Loneliness and its effect on meditation is a topic of growing concern; not only has this topic gained more traction due to the growing demographic of aging populations experiencing loneliness, but there is also a public concern about how this demographic will burden the healthcare system and affect public finances [37]. Since loneliness leads to negative health outcomes and is disproportionately seen in the elderly, a focus on how meditation can alleviate loneliness can further reduce the burden on healthcare systems by having these individuals lead more healthy aging lives [36]. With mediation working to alleviate feelings of loneliness, this can result in various positive health outcomes, such as a lower risk for physiological dysregulation and inflammation [18]. Inflammation, specifically, can lead to the development of various diseases that can cause late-life morbidity and mortality, therefore, lowering one’s risk of inflammation through the alleviation of loneliness can have significant benefits for one’s foreseeable future [9].

It is worth noting that even in many of the ineligible full texts that we had read (but ultimately excluded from this review), social closeness and social isolation were also key focus areas, rather than loneliness, in relation to meditation. Social isolation and loneliness are terms that are often used interchangeably within the literature; we argue that these terms are different, however, as individuals experiencing loneliness may or may not be be socially isolated, and socially isolated people are not always experiencing loneliness [41]. Loneliness is based on how a person feels about their social situation, whereas social isolation is a state regarding one’s social situation (i.e. lack of proximity to others). Loneliness also exists in relation to the perceived lack of connectedness felt from interpersonal relationships, which is why social closeness is often a measure of loneliness [41]. Similar to social isolation, social closeness is a state of being around others, though this does not necessarily mean one does or does not feel lonely. The development of a universally agreed-upon definition of loneliness is also warranted to better standardize research aiming to evaluate the value of therapies in alleviating this condition. With this in mind, we have identified a number of warranted directions for future research which could build on the present review’s findings.

### Areas identified for future research

Relatively small sample sizes were reported across all 13 eligible studies. In line with future directions proposed in some of these eligible studies, a need exists to conduct larger scale research studies to better understand the impacts of meditation on loneliness. Goyal et al. [42] conducted a systematic review and found that meditation programs were useful in reducing psychological stress and showed small improvements for conditions such as anxiety, depression, and pain. However, similar to our scoping review, all of their included studies had small sample sizes ranging from 15 to 201 patients, which supports this identified need for studies including larger sample sizes. Additionally, across all eligible articles, there was no consistent or unified method for measuring participants’ degree of loneliness nor its alleviation; instead, we found 4 methods of measurement across the 13 studies. Eight of the 13 articles (61%) utilized the UCLA Loneliness Scale, while other studies used the DJGLS-6 (n = 1) or their own variation of measurement (i.e. baseline and post-treatment surveys, and self-reported questionnaires) (n = 2). Hughes et al. [43] have described the Revised UCLA Loneliness Scale as a long, complex self-administered scale with 20 items and four response categories each. The participant’s responses are summed up, and a higher score indicates greater loneliness, making the scale less suitable for telephone surveys in large-scale studies [43]. While this scale is considered to be the most psychometrically sound and most frequently used measure for loneliness, it has been criticized for only measuring the social dimension of loneliness, as opposed to the emotional dimension [44]. In contrast, the Three-Item Loneliness Scale gauges general feelings of loneliness well and can be used across two interview modalities (in-person self-administered and telephone), with the possibility existing for this scale to be embedded within the Revised-UCLA (R-UCLA) itself [43]. In comparison to these methods of measurement, the DJGLS-6 was developed more recently in 2006, taking into account both emotional and social dimensions, but has been less well-evaluated than the R-UCLA [44]. There also appears to be a need to conduct further research that utilizes recently created modes of measurement, such as the DJGLS-6, in order to better evaluate its overall use, reliability, and validity [44]. This could serve to inform the creation of a widely accepted measurement of loneliness that assesses both emotional and social dimensions, which could be used across various interview modalities. Greater knowledge of the specific and targeted benefits (and risks and side effects) of meditation in the context of alleviating loneliness can better assist clinicians in facilitating shared decision making with their patients regarding these interventions [42].

It is also worth noting that the majority of included studies either did not collect or report on the socioeconomic status of their participants (69%). Those that did report it, generally had a lack of study participants identifying as belonging to a lower-socioeconomic class and/or without a high school education, therefore, it is unclear whether findings are applicable to these populations. Thus, a need exists for future studies in this area to include participants from diverse backgrounds in order to account for populations which have been understudied, as such individuals may suffer disproportionately from loneliness.

### Strengths and limitations

Notable strengths of this study included the use of a comprehensive systematic search strategy to identify eligible articles. Interpretation of these findings was strengthened by the fact that three authors (GKS, SBH, and ZT) independently screen, data extract, and summarize findings, with assistance from the supervising author (JYN). Limitations include the fact that this scoping review did not include non-English language articles, therefore, studies emerging from non-English speaking countries may not have been captured.

## Conclusions

The present scoping review involved a systematic search of the literature to identify the quantity and type of studies investigating the effects of meditation on loneliness. From 13 eligible articles, we identified three major themesincluding: 1) positive results across all studies, 2) relatively small randomized control trials conducted over the last decade, and 3) lack of diverse demographic information. While a small number of studies exist at this intersection, given that all included studies reported positive findings, the effects of meditation in alleviating loneliness are promising. Based on our findings, future studies should consider the use of newer modes of measurement for loneliness, such as the DJGLS-6 and continue to evaluate the utility of meditation in alleviating loneliness; this is especially warranted as the COVID-19 pandemic progresses. Future research should also involve larger sample sizes with a range of participants from various backgrounds and be directed at improving our understanding of how meditation serves to alleviate loneliness.

## Data Availability

The datasets used and/or analysed during the current study are available from the corresponding author on reasonable request.

## Abbreviations

DJGLS-6: 6-Item de Jong Gierveld Loneliness Scale
NF: Nuclear factor
RCT: Randomized control trial
R-UCLA: Revised UCLA
